# Aedes aegypti in Tucson, Arizona.

**DOI:** 10.3201/eid0404.980428

**Published:** 1998

**Authors:** T M Fink, B Hau, B L Baird, S Palmer, S Kaplan, F B Ramberg, D G Mead, H Hagedorn


### *Aedes aegypti* in Tucson, Arizona

**To the Editor**: The highly domestic mosquito species *Aedes aegypti,* a tropical, nonnative vector of dengue and yellow fever, has been identified in several desert communities, including the city of Tucson and the border towns of Douglas, Naco, and Nogales (1). *Ae. aegypti* has now been found in the southern Arizona communities of Benson and Sahuarita Heights, which indicates that its distribution is probably expanding. Moreover, mosquito surveillance indicating that *Ae. aegypti* populations in Tucson are established throughout the city supports local concerns that the mosquito poses a public health risk in Arizona's second largest metropolitan area.

Since it was first detected in 1994 on Tucson's west side, the mosquito has been found in the central downtown and university districts, northern foothills, and the east and south sides (both in residential areas, including schools and parks, and in business districts). Between 1995 and 1997, almost 200 adult *Ae. aegypti* (female and male) were recovered in CDC $CO_2$ traps from 26 sites in Tucson by vector biologists from the Arizona Department of Health Services, Pima County, and the University of Arizona Veterinary Science Department.

These trapping events occurred between May and October of each year and were associated with either routine arbovirus surveillance or nonsystematic *Ae. aegypti* "spot-checks." Many of the sites were sampled more than once and 30% contained *Ae. aegypti* on multiple occasions. Catches from individual sites contained 1 to 40 adult mosquitoes (mode = 1).

A larval survey of the city has not yet been conducted because of limited staff and problems associated with oviposition trapping, an important part of *Ae. aegypti* larval surveillance (2). Initial attempts at ovitrapping by state, county (1), and later university personnel were not successful because the hay infusion water in ovitraps evaporated rapidly in Tucson's arid climate. However, the few larvae recovered from household containers suggest that Tucson's urban environment is providing a breeding habitat. Since the local climate requires full-time vigilance of ovitraps, this method of surveillance appears too labor intensive for the present.

In 1997, the University of Arizona Entomology Department initiated a mosquito survey of the city. This multiyear project is funded by the Entomology Department, the city of Tucson, and the Pima County Health Department. Neighborhood associations in Tucson were surveyed for their perceptions of the magnitude of the mosquito problem in their areas. On the basis of survey results, the city was divided into regions: north, east, south, west, and central. Four trapping stations were established in each region for a total of 20 sites spanning the metropolitan Tucson and outlying areas. The five regions were surveyed for mosquitoes through use of $CO_2$ traps approximately every 10 days starting July 1, 1997; traps were set in the late afternoon and collected in the late morning. Daytime $CO_2$ trappings were not effective.

The Entomology Department's 1997 surveillance data suggest that the central part of the city is the most heavily infested. Of 95 adult *Ae. aegypti* trapped, 49.5% were from the central region of Tucson, 18.9% from the west side, 17.9% from the east side, 10.5% from the north side, and 3.2% from the south side. The mosquito populations appeared to fluctuate with the weather, increasing in size after rainfall. Long-term trapping and future larval surveys should shed more light on this association.

Pima County and the University of Arizona Veterinary Science Department trapping activities in 1997 also produced evidence of *Ae. aegypti* in two communities near Tucson. Six adult mosquitoes were recovered in the town of Benson, 30 miles southeast of Tucson, and seven were trapped in Sahuarita Heights, 15 miles south of Tucson. The presence of the mosquitoes in these communities, as well as in Douglas, Naco, and Nogales, demonstrates that Arizona's smaller desert communities are also susceptible to *Ae. aegypti* infestations. The humidity emitted by home evaporative coolers may be crucial for the survival of tropical mosquitoes, such as *Ae. aegypti,* in Arizona's arid climate (N. Monteny, pers. comm.).

Genetic analysis of the *Ae. aegypti* collected from southeastern Arizona, Texas, and Mexico is under way at the University of Arizona Ecology and Evolutionary Biology Department to determine the structure, history, and origin(s) of the reemergent mosquito populations. Preliminary findings from mitochondrial DNA sequences suggest that *Ae. aegypti* in Arizona represent a single (panmictic) population, which indicates frequent local migration. More extensive sampling is necessary to confirm these results and determine a point of origin.

A community outreach program has been developed to inform the public about *Ae. aegypti* breeding and control in Tucson. Public involvement will be a key factor in the control of these urban breeders. Major emphasis will also be placed on programs for children and teachers as both groups can be instrumental in maintaining long-term interest in this problem. As these programs are developed, they can be expanded and amended to meet the needs of other infested communities in southern Arizona. A mosquito control abatement district is under consideration in a central part of Tucson. The primary purpose of this district would be to provide approximately 10,000 homeowners with information on controlling *Ae. aegypti* breeding on their property.

Just how long the *Ae. aegypti* infestation will last is difficult to assess. Records of the city's earlier infestation indicate the mosquito was present for at least a 15-year period (1931 to 1946) (1,3,4). Since their identification in early 1998 summer mosquito samples from Tucson, adult *Ae. aegypti* have been part of the city's local environment for at least 5 consecutive years (1994 to 1998). Their continued presence and the abundant breeding habitat provided by the expansion of Tucson's urban landscape suggest that *Ae. aegypti* could survive for an extended period.

**T. Michael Fink,* Bosun Hau,† Branford L. Baird,‡ Sarah Palmer,† Susanne Kaplan,† Frank B. Ramberg,† Daniel G. Mead,† and Henry Hagedorn†**
*Arizona Department of Health Services, Phoenix, Arizona, USA; †University of Arizona, Tucson, Arizona, USA; and ‡Pima County Community Prevention and Public Health Department, Tucson, Arizona, USA

### References

1. Engelthaler DE, Fink TM, Levy CE, Leslie MJ. The reemergence of *Aedes aegypti* in Arizona. Emerg Infect Dis 1997;3:241-2.
2. Reiter P, Amador MA, Colon N. Enhancement of the CDC ovitrap with hay infusions for daily monitoring of *Aedes aegypti* populations. J Am Mosq Control Assoc 1991;7:52-5
3. Murphy D. Collection records of some Arizona mosquitoes. Entomological News 1953;14:233-8.
4. Bequaert J. *Aedes aegypti*, the yellow fever mosquito, in Arizona. Bulletin of the Brooklyn Entomological Society 1946;41:157.

### Can the Military Contribute to Global Surveillance and Control of Infectious Diseases?

**To the Editor:** Numerous networks—both formal (e.g., Ministries of Health and WHO Collaborating Centers and collaborating laboratories) and informal (e.g., nongovernmental and humanitarian organizations, the media, and electronic discussion groups)—contribute to WHO's network of networks for the global surveillance of infectious diseases (1).

A potential source of additional information on infectious diseases is the network of military health facilities and laboratories throughout the world. In addition to health facilities serving populations at high risk for infectious diseases, the military also has laboratories, often among the better-equipped, in developing countries. To evaluate the feasibility and potential usefulness of including military laboratories in the WHO global surveillance network, we conducted three surveys.

The first survey identified military laboratories willing to participate in global surveillance activities and obtained information about their infectious diseases reporting systems. Of the 107 countries surveyed, 76 replied. Among them, 53 (70%) reported having a central military laboratory that coordinates laboratory activities throughout the military, and 62 (82%) reported that military clinical facilities had a reporting system for infectious diseases.

The second survey quantified laboratory capabilities in the 53 laboratories identified in the first survey and obtained details about the 62 reporting systems. Among the 39 (74%) laboratories that replied, all can perform at least one of the following activities: isolating and identifying bacterial, viral, or parasitic agents. Twenty-nine (55%) have the capacity for specialized immunologic or molecular study. In addition, one of these laboratories has a biosafety level 4 facility, six have a biosafety level 3 facility, and 10 have a biosafety level 2 facility. Twenty-seven (51%) of the laboratories perform compulsory screening of new recruits for HIV, 17 (33%) for hepatitis B, 7 (13%) for hepatitis C, 39 (74%) for tuberculosis, 35 (67%) for syphilis, 18 (34%) for intestinal parasites, 13 (25%) for schistosomiasis, 12 (23%) for malaria, and 2 (4%) for Chagas disease.

